# Does obstructive sleep apnoea modulate cardiac autonomic function in paroxysmal atrial fibrillation?

**DOI:** 10.1007/s10840-022-01202-3

**Published:** 2022-04-09

**Authors:** Anna Mary Mohammadieh, Hasthi U. Dissanayake, Kate Sutherland, Seren Ucak, Philip De Chazal, Peter A. Cistulli

**Affiliations:** 1 Sleep Research Group, Charles Perkins Centre and Northern Clinical School, Faculty of Medicine and Health, The University of Sydney, Sydney, Australia; 2 Department of Respiratory and Sleep Medicine, Centre for Sleep Health & Research, Level 8, Acute Services Building, Royal North Shore Hospital, St Leonards, NSW 2065 Australia; 3 Department of Thoracic Medicine, St Vincent’s Hospital, Darlinghurst, 2010 Australia; 4 School of Biomedical Engineering, University of Sydney, Sydney, NSW 2006 Australia

**Keywords:** Heart rate variability, Cardiac autonomic function, Atrial fibrillation, Obstructive sleep apnoea

## Abstract

**Purpose:**

The autonomic nervous system may mediate acute apnoea–induced atrial fibrillation (AF). We compared cardiac autonomic function in paroxysmal atrial fibrillation (PAF) patients with and without obstructive sleep apnoea (OSA).

**Methods:**

Case control study of 101 patients with PAF recruited at two tertiary centres. All patients underwent in-laboratory polysomnography. ECG signal demonstrating “steady state” sinus rhythm (i.e. with arrhythmic beats and respiratory events excluded) was included in the analysis. Cardiac autonomic function was assessed via measures of heart rate variability (HRV) and reported by sleep stage (REM vs Non-REM) for patients with and without OSA.

**Results:**

Sixty-five (66.3%) of patients were male, mean age 61.5 ± 11.6 years, mean BMI 27.1 ± 4.3 kg/m^2^. Global measures of HRV (triangular index, total power) did not differ between PAF patients with and without OSA in either REM or non-REM sleep. Frequency-domain analysis during non-REM sleep in PAF patients with OSA showed increased cardiac parasympathetic modulation (HF-nu: 39.1 ± 15.7 vs 48.0 ± 14.6, *p* = 0.008) and reduced cardiac sympathetic modulation (LF-nu 54.1 ± 19.7 vs 43.7 ± 18.0, *p* = 0.012, LF/HF ratio: 2.1 ± 2.0 vs 1.2 ± 1.0, *p* = 0.007). Results remained significant after adjusting for age, sex, and BMI (adjusted *p* values 0.024, 0.045 and 0.018 respectively). There were no differences in HRV parameters during REM sleep.

**Conclusions:**

This is the first study of HRV in PAF patients with and without OSA. Our results indicate limited differences in HRV between groups. However, this work suggests a chronic increase in parasympathetic nervous modulation and relative reduction in sympathetic modulation in PAF patients with OSA during steady-state non-REM sleep.

**Supplementary Information:**

The online version contains supplementary material available at 10.1007/s10840-022-01202-3.

## Introduction

Atrial fibrillation (AF) is the most common sustained cardiac arrhythmia and is associated with increased risk of stroke and congestive heart failure [1]. Mounting evidence suggests that dysregulation of the cardiac autonomic axis plays an integral role in arrhythmogenesis [2].

OSA is a highly prevalent sleep disorder characterised by upper airway collapse during sleep and is found in up to 63% of AF patients [3]. Attempting to breathe against an obstructed upper airway results in intermittent hypoxia, intra-thoracic pressure swings and activation of the autonomic nervous system; these acute perturbations are thought to trigger and maintain episodes of AF [4, 5], and may lead to long-term atrial remodelling [5, 6].

Understanding the influence of OSA on autonomic function in patients with AF may inform treatment strategies that mitigate pro-arrhythmic autonomic influences. Heart rate variability (HRV) reflects beat-to-beat variation in heartbeat intervals influenced by the combined effects of the sympathetic and parasympathetic nervous system [7]. The study of HRV provides a non-invasive method to assess cardiac autonomic function [7]. We aimed to assess whether in a paroxysmal atrial fibrillation (PAF) cohort the presence of OSA is associated with altered autonomic function. We hypothesised that PAF patients with OSA will show altered HRV parameters indicative of the influence of OSA on cardiac autonomic function.

## Methods

### Study population

Sequential AF patients were recruited via two centres as part of a prospective diagnostic accuracy study for sleep apnoea in patients with AF [3]. Approval for this trial was obtained from the Northern Sydney Local Health District Human Research Ethics Committee (HREC/16/HAWKE/25) and the North Shore Private Hospital Ethics Committee (approval number 2016–012). The study was performed in accordance with the 1964 Helsinki Declaration and its later amendments. All the patients gave their informed written consent to participate in the study. The trial was registered with the Australian New Zealand Clinical Trials Registry (ANZCTR): 12,616,001,016,426.

The patients were sequentially recruited via two pathways: emergency department admissions with AF and pulmonary vein isolation waitlists at two tertiary centres between July 2016 and September 2019. All the patients had a history of AF (≥ 2 episodes in the past 12 months) and underwent in-laboratory polysomnography (PSG) to investigate OSA. The patients with a previous known diagnosis of sleep apnoea were excluded.

### Data collection

#### Polysomnography

Polysomnographic recordings were performed and scored by experienced sleep scientists using Compumedics PSG4 V4.1 software (Compumedics, Australia), according to the American Academy of Sleep Medicine (AASM) criteria [8]. An apnoea was defined as complete (≥ 90%) reduction in airflow, lasting ≥ 10 s. A hypopnea was defined as a partial (≥ 30%) reduction in airflow, lasting ≥ 10 s, associated with either an arousal from sleep or an oxygen desaturation of ≥ 3% from baseline. Sleep apnoea was defined as the average number of apnoea and/or hypopneas per hour (apnoea hypopnea index (AHI)) ≥ 5/h. Other standard parameters of OSA severity were generated including ODI (oxygen desaturation index): average number of desaturations per hour > 3% below baseline, and %T < 90: the percent of sleep time with SaO_2_ < 90%. OSA severity was defined as mild (AHI ≥ 5–14.9/h), moderate (AHI ≥ 15–29.9/h) and severe (AHI ≥ 30/h).

#### Holter monitor processing

Electrocardiographic (ECG) signals from the polysomnographic recording were processed using Holter software analysis for ectopic beat detection (SpaceLabs Sentinel v11.5.1.12779 and Pathfinder SL version 1.9.2.11104, Snoqualmie, WA 98,065, USA). Three traces were excluded as the ECG quality was insufficient for Holter software analysis. Since heart rate variability (HRV) is ordinarily performed during sinus rhythm, six traces were excluded as patients were in AF for the vast majority (≥ 90%) of the study night. The patients with shorter runs of AF were included, though the periods of AF were excluded from the analysis (three patients, with 1.8, 22.4 and 3.1% of the night spent in AF, respectively).

#### Heart rate variability analysis

**Fig. 1:**
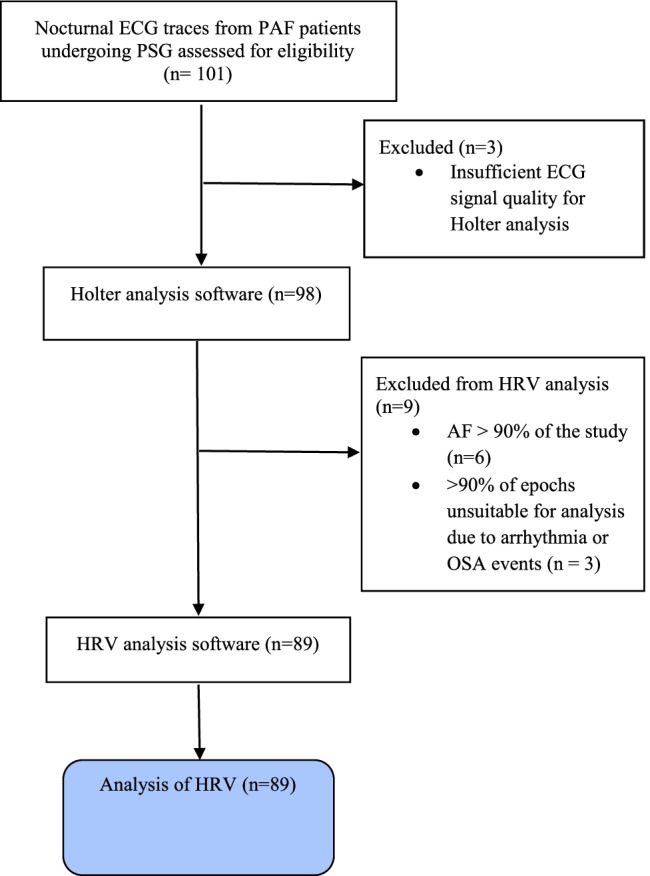
Study flowchart

Following Holter analysis, ECG signals were analysed following the guidelines of Task Force of the European Society of Cardiology and the North American Society of Pacing and Electrophysiology [7]. QRS detection was performed to a resolution of 1 ms (equivalent to a sample rate of 1000 Hz). HRV analysis was performed using a validated algorithm [9] using MATLAB 2017, version 9.2.0.538062 (R2017a), Natick, Massachusetts: The MathWorks Inc. analysis was performed over 2-min epochs averaged across each sleep stage (NREM and REM) of the entire ECG signal. Epochs with mixed sleep stages were excluded, as were periods of arousal, apnoeas, hypopneas, respiratory event-related arousals as well as artefact as per previously published [10–12]. A 15-s interval following obstructive respiratory events was excluded to control for acute post-event autonomic perturbations. Furthermore, periods of cardiac arrhythmia including atrial and ventricular ectopic beats were excluded from the HRV analysis (2.65% of total beats). If the excluded periods of an epoch exceeded 12 s (10% of epoch length), then the complete epoch was excluded. The patients were excluded from further analysis if > 90% of 2-min epochs were excluded on the basis of arrhythmia or sleep apnoea events (3 patients). A total of 89 studies were therefore included in the HRV analysis (Fig. 1).

#### Time and frequency-domain measures of HRV

Time-domain measures of HRV provide the simplest of method to evaluate variations in heart rate. These include (a) HRV triangular index (HRVi) indicative of overall HRV, (b) RMSSD which is the root mean square of successive differences in NN intervals and (c) pNN50, the proportion of successive NN intervals which differ by more than 50 ms. Both RMSSD and pNN50 are short-term measures and are estimates of high frequency variations in heart rate; these measures reflect parasympathetic modulation of the heart [7, 13].

Frequency-domain measures of HRV provide information on sympathetic and parasympathetic modulation of beat-to-beat fluctuations in the heart rate. An analysis was performed using a Lomb periodogram method, and the spectral bands for HRV were investigated in the range of 0–0.4 Hz: very low frequency (VLF) at 0 to 0.04 Hz, low frequency (LF) at 0.04–0.15 Hz and high frequency (HF) power at 0.15 to 0.4 Hz [7]. LF and HF were also expressed in normalised units, LF-nu and HF-nu, respectively [7]. The HF component of HRV, synchronous with respiration, is considered a strong marker of parasympathetic modulation [7]. The interpretation of the LF component of HRV is more controversial; however, when expressed in normalised units (nu), it is a marker of sympathetic modulation. We have applied this methodology in evaluating the level of sympathetic and parasympathetic modulation at the atrial site [7]. A summary of time and frequency domain–HRV variables is provided in Table 1.

**Table 1:** Summary of time and frequency domain–HRV parameters

HRV parameter (units)	Description
*Time domain*	
NN interval (ms)	Interval between R wave peaks for two normal successive beats
Average NN interval (ms)	Average time interval between normal successive R waves
RMSSD (ms)	Square root of the mean squared differences of successive NN intervals
pNN50 (%)	Proportion of NN intervals which differ by more than 50 ms
Triangular index (nu)	Integral of the density of the RR interval histogram divided by its height, a measure of total HRV
*Frequency domain*	
High frequency (ms^2^)	Power in the 0.15–0.40-Hz band
High frequency–normalised units (%)	HF power divided by power ≥ 0.04 Hz
Low frequency (ms^2^)	Power in the 0.04–0.15-Hz band
Low Frequency–normalised units (%)	LF power divided by power ≥ 0.04 Hz
Very low frequency (ms^2^)	Power less than 0.04 Hz
LF/HF ratio (nu)	Low frequency power divided by high frequency power
Total power	Power from 0 to Nyquist frequency

### Statistical analysis

Statistical analysis was performed using IBM SPSS Statistics for Windows, Version 27.0, Armonk, NY: IBM Corp. HRV parameters which were not normally distributed were natural-log transformed to normalise their skewed distributions. Data are presented as the mean + / − standard deviation (SD), or as mean (interquartile range (IQR)) for non-normally distributed data. HRV parameters were compared using *t*-tests (continuous variables) or chi-square tests (categorical variables) as appropriate. Analysis of covariance (ANCOVA) was performed to adjust for age, sex and BMI. Correlations between HRV parameters and standard measures of OSA severity (AHI, ODI and T < 90%) were performed. This was an exploratory analysis of the comparison of multiple HRV parameters reflecting different aspects of ANS modulation; therefore, we did not adjust the significance level for multiple comparisons. We considered a *p* value of < 0.05 statistically significant.

## Results

### Patient characteristics

A total of 101 AF patients underwent overnight polysomnography. The patients with OSA demonstrated a higher BMI and were predominantly male compared to the no-OSA group. They also had increased hypertension but reduced thyroid disease and peripheral vascular disease. There were no significant differences in other cardiovascular comorbidities including diabetes, ischaemic heart disease (IHD), congestive cardiac failure (CCF) and cerebrovascular disease (CVD), although the OSA group did have a lower ejection fraction (55.5 ± 9.7 vs 59.6 ± 1.1%, *p* = 0.027). The OSA group also had an increased left atrial area (25.5 ± 5.3 vs 22.3 ± 4.5 cm^2^, *p* = 0.032) and increased proportion of “high burden AF”, defined as > 12 self-reported episodes in the last 12 months. CHA_2_DS_2_-Vasc scores were not significantly different between the groups (1.4 ± 1.2 vs 1.7 ± 1.4, *p* = 0.270). Neither were there significant differences in anti-arrhythmic medications between groups. Although nine patients were excluded from the final HRV analysis, excluded patients were not significantly different from the group in terms of key baseline characteristics including age, sex and BMI. Baseline characteristics are presented in Table 2.

**Table 2:** Baseline characteristics of AF patients with and without OSA (AHI ≥ 5/h)

Characteristic	*N* (%) or mean ± SD			
	Mean (SD) or *n* (%) *n* = 98	AHI < 5/h *n* = 36	AHI ≥ 5/h *n* = 62	*p* value
*General demographics*				
Recruitment stream: ER	54 (55.1)	19 (52.8)	35 (56.5)	0.834
Age (years)	61.5 ± 11.6	58.9 ± 12.5	63.0 ± 10.8	0.098
Male	65 (66.3)	19 (52.8)	46 (74.2)	0.045
Ethnicity: Caucasian	91 (92.9)	34 (94.4)	57 (91.9)	0.384
*Phenotypic characteristics*				
BMI (kg/m^2^)	27.1 ± 4.3	24.7 ± 3.5	28.5 ± 4.2	< 0.001
Neck circumference (cm)	40.0 ± 4.9	38.8 ± 4.7	40.7 ± 4.9	0.065
Modified Mallampati score	2.7 ± 0.9	2.4 ± 0.9	2.8 ± 0.8	0.028
*Co-morbidities/AF risk factors (n* = *99)*				
Alcohol excess (≥ 10 SD/week), *n* = 105	21 (21.4)	12 (33.3)	11 (17.8)	0.136
Thyroid disease	16 (16.3)	11 (30.6)	5 (8.1)	0.009
Family history of AF	29 (29.6)	13 (36.1)	16 (25.8)	0.203
Mod-severe MS/prosthetic heart valve	3 (3.0)	2 (2.9)	1 (3.1)	0.865
Hypertension	38 (38.8)	8 (22.2)	31 (50.0)	0.010
Diabetes	4 (4.1)	2 (5.6)	3 (4.8)	0.876
IHD	4 (4.1)	1 (2.8)	3 (8.3)	0.619
CCF	18 (18.4)	5 (13.8)	13 (21.0)	0.431
Cerebrovascular disease	2 (2.0)	0 (0)	2 (3.2)	0.530
Peripheral vascular disease	3 (3.1)	3 (8.3)	0 (0)	0.047
CHA_2_DS_2_-Vasc score	1.6 ± 1.3	1.4 ± 1.2	1.7 ± 1.4	0.270
Anti-coagulant therapy	78 (79.6)	30 (83.3)	49 (79.0)	0.792
*AF characteristics*				
Paroxysmal	90 (91.8)	36 (100.0)	57 (91.9)	0.080
High burden (≥ 10 episodes AF last 12 M)	31 (31.6)	8 (22.2)	23 (37.1)	0.039
Prior PVI (number)	0.4 ± 0.6	0.33 ± 0.05	0.40 ± 0.64	0.570
Prior cardioversion (number)	1.0 ± 1.5	0.64 ± 1.18	1.16 ± 1.57	0.086
*Anti-arrhythmic therapy*				
Prescribed anti-arrhythmic medication?	78 (79.6)	30 (83.3)	50 (80.6)	0.794
Number of anti-arrhythmic medications	1.05 (0.6)	1.06 (0.63)	1.06 (0.67)	0.948
Class 1 anti-arrhythmic	29 (29.6)	11 (30.6)	18 (29.0)	0.873
Class 2 anti-arrhythmic	30 (30.6)	11 (30.6)	19 (30.6)	0.993
Class 3 anti-arrhythmic	28 (28.6)	9 (0.25)	19 (30.6)	0.646
Class 4 anti-arrhythmic	9 (9.2)	3 (8.3)	6 (9.7)	0.824
Class 5 anti-arrhythmic	3 (3.1)	1 (2.8)	2 (3.2)	0.901
*Echocardiographic parameters (n* = *74)*				
Cardiac ejection fraction (%)	57.1 ± 8.7	59.6 ± 1.1	55.5 ± 9.7	0.027
Left atrial diameter (cm) (*n* = 53)	4.1 ± 6.3	3.9 ± 0.5	4.2 ± 0.7	0.105
Left atrial area (cm^2^) (*n* = 46)	24.2 ± 5.2	22.3 ± 4.5	25.5 ± 5.3	0.032
*OSA symptoms*				
ESS	6.2 ± 3.4	5.7 ± 3.8	6.4 ± 3.1	0.349
Self-reported snoring	64 (65.3)	18 (50.0)	46 (74.2)	0.027
*Sleep parameters from PSG*				
AHI (/h)	13.8 ± 15.9	1.7 ± 1.4	20.8 ± 16.3	< 0.001
ODI (/h)	7.4 ± 10.9	0.8 ± 1.5	11.2 ± 12.2	< 0.001
CAI (/h)	0.6 ± 1.5	0.1 ± 1.2	0.8 ± 1.8	0.018
AI (/h)	2.4 ± 5.3	0.2 ± 0.3	3.7 ± 6.3	< 0.001
HI (/h)	11.4 ± 13.2	1.5 ± 1.4	17.1 ± 13.8	< 0.001
*Presence of arrhythmia on PSG*				
AF (% of total beats)	6.2 ± 23.4	2.9 ± 16.6	8.1 ± 26.4	0.283
SVEB (% of total beats)	0.6 ± 1.6	0.9 ± 2.0	0.5 ± 1.4	0.136
VEB (% of total beats)	0.5 ± 1.7	0.5 ± 1.4	0.4 ± 1.8	0.826

*AF* atrial fibrillation, *AHI* apnoea hypopnea index, *BMI* body mass index, *CAI* central apnoea index, *CCF* congestive cardiac failure, *ER* emergency room, *ESS* Epworth Sleepiness Scale, *IHD* ischaemic heart disease, *MS* mitral stenosis, *ODI* oxygen desaturation index, *PVI* pulmonary vein isolation procedure waitlist, *SD* standard drinks, *SVE* supraventricular ectopic beats, *VE* ventricular ectopic beats

### Atrial fibrillation characteristics

**Fig. 2:**
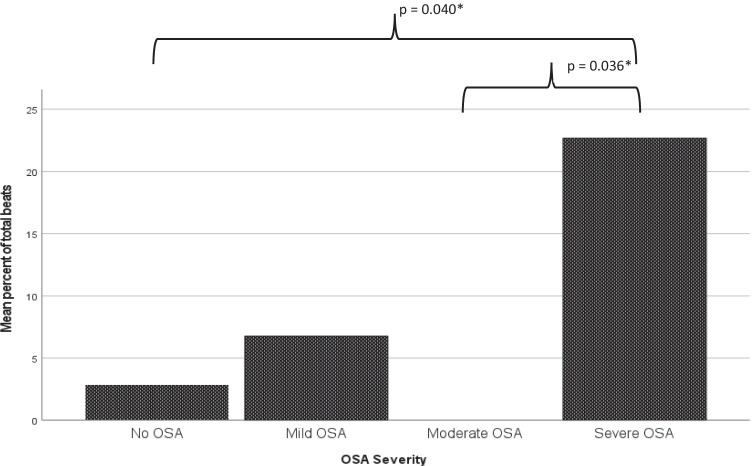
AF beats as a percent of total beats by OSA severity. Patients with no OSA (AHI ≤ 5/h, *n* = 36) had 2.9 ± 16.6% AF beats; patients with mild OSA (AHI 5–14.9/h, *n* = 31) had 6.7 ± 23.6% AF beats; patients with moderate OSA (AHI 15–29.9/h, *n* = 18) had 0.0 ± 0.0% AF beats, and those with severe OSA (AHI ≥ 30/h, *n* = 13) had 22.7 ± 42.8% AF beats

There were no significant differences in the presence of arrhythmia (AF beats, ventricular ectopic beats (VEBs) and supraventricular ectopic beats (SVBs)) between AF patients with and without OSA using a cut-off AHI of 5/h (see Table 2). On subgroup analysis, however, PAF patients with severe OSA (AHI ≥ 30/h) had more AF beats and ventricular ectopic beats (VEBs) than those without severe OSA (22.7 ± 42.8% vs 3.7 ± 17.9%, *p* = 0.006, 1.7 ± 3.8 vs 0.3 ± 0.9%, *p* = 0.004, respectively). Similarly, ANOVA for OSA severity groups showed that PAF patients with severe OSA had a higher % AF beats than patients with no OSA or moderate OSA (mean difference 19.8 ± 7.3%, *p* = 0.040; 22.7 ± 8.3%, *p* = 0.036 respectively, see Fig. 2) and that PAF patients with severe OSA had a higher %VEBs than patients with mild or moderate OSA (mean difference 1.6 ± 0.5%, *p* = 0.019; 1.6 ± 0.6%, *p* = 0.035, respectively). There were no significant differences in SVEs across OSA severity groups.

### Heart rate variability in PAF patients with and without OSA

In non-REM sleep, time-domain measures of HRV did not differ between OSA and no-OSA groups (Table 3). However, we saw selective differences in frequency-domain measures (Table 4). Specifically, PAF patients with OSA showed increased parasympathetic modulation (HF-nu: 48.0 ± 14.6 vs 39.1 ± 15.7, *p* = 0.01) and reduced sympathetic modulation (LF-nu 43.7 ± 18.0 vs 54.1 ± 19.7, *p* = 0.01). Consistent with these results, the LF/HF ratio showed a relative decrease in parasympathetic modulation (1.2 ± 1.0 vs 2.1 ± 2.0, *p* = 0.007) (Table 4). Furthermore, these results remained significant after adjusting for age, sex and BMI.

**Table 3:** HRV time–domain parameters by OSA status in a cohort with atrial fibrillation

	All patients	No OSA AHI < 5/h	OSA AHI > 5/h	*p* value	*p* value (adjusted for age, sex, BMI)
	*n* = 89	*n* = 35	*n* = 54		
Average NN interval (ms)					
Non-REM	1080.2 ± 162.9	1062.0 ± 133.9	1091.9 ± 179.4	0.401	0.475
REM	1067.2 ± 166.4	1041.2 ± 136.7	1084.3 ± 182.5	0.243	0.492
†RMSSD (ms)					
Non-REM	28.7 (23.1)	27.3 (20.7)	30.9 (23.6)	0.447	0.570
REM	26.7 (22.7)	20.4 (20.8)	25.3 (23.5)	0.376	0.374
†pNN50 (%)					
Non-REM	5.5 (14.7)	4.5 (12.2)	6.2 (15.9)	0.063	0.061
REM	2.8 (12.3)	1.9 (11.1)	3.3 (17.2)	0.772	0.631
Triangular index (nu)					
Non-REM	10.8 ± 4.3	10.7 ± 4.4	10.8 ± 4.4	0.975	0.612
REM	11.7 ± 4.6	11.7 ± 4.6	11.6 ± 4.7	0.885	0.541

Data are presented as mean ± SD or median (IQR). Natural log-transformed data are indicated by †

*Non-REM* non-rapid eye movement, *REM* rapid eye movement, average NN interval average of N wave to N wave variation, *RMSSD* square root of the mean squared differences of successive NN intervals, *pNN50* percentage of successive NN intervals that differ by more than 50 ms, triangular index*:* integral of the density of the RR interval histogram divided by its height

**Table 4:** HRV frequency–domain parameters by OSA status in a cohort with atrial fibrillation

	All patients	No OSA AHI < 5/h	OSA AHI > 5/h	*p* value	*p* value (adjusted for age, sex, BMI)
	*n* = 89	*n* = 35	*n* = 54		
†High frequency (ms^2^)					
Non-REM	333.3 (437.8)	238.8 (913.9)	403.5 (723.7)	0.643	0.730
REM	223.3 (437.8)	206.6 (344.1)	239.8 (531.1)	0.530	0.426
High frequency–normalised units (%)					
Non-REM	44.5 ± 15.6	39.1 ± 15.7	48.0 ± 14.6	0.008	0.024
REM	37.4 ± 17.2	32.6 ± 16.0	40.5 ± 17.4	0.036	0.143
†Low frequency (ms^2^)					
Non-REM	336.1 (863.9)	339.6 (1155.7)	327.6 (760.1)	0.447	0.559
REM	302.9 (817.5)	358.5 (1103.7)	298.8 (831.0)	0.533	0.904
Low frequency–normalised units (%)					
Non-REM	47.8 ± 19.2	54.1 ± 19.7	43.7 ± 18.0	0.012	0.045
REM	55.2 ± 22.1	60.4 ± 20.8	51.8 ± 22.5	0.076	0.228
†Very low frequency (ms^2^)					
Non-REM	470.0 (860.0)	603.8 (1055.1)	445.2 (605.6)	0.135	0.148
REM	865.4 (1322.0)	954.9 (1548.9)	826.5 (1307.6)	0.417	0.663
LF/HF ratio (nu)					
Non-REM	1.5 ± 1.5	2.1 ± 2.0	1.2 ± 1.0	0.007	0.018
REM	2.3 ± 2.2	2.9 ± 2.6	2.0 ± 1.9	0.063	0.108
†Total power					
Non-REM	1352.7 (2078.9)	1183.2 (1514.3)	1109.0 (1573.3)	0.526	0.987
REM	1128.2 (1498.7)	1439.0 (2360.6)	1318.0 (2137.8)	0.666	0.988

Data are presented as mean (SD) or median (IQR). Natural log-transformed data are indicated by †. High frequency: power in the 0.15–0.40 Hz band; high frequency–normalised units: HF power divided by power ≥ 0.04 Hz; low frequency: power in the 0.04–0.15 Hz band; low frequency–normalised units: LF power divided by power ≥ 0.04 Hz; very low frequency: power less than 0.04 Hz; LF/HF, low frequency/high frequency ratio: low frequency power divided by high frequency power; total power: power from 0 to Nyquist frequency

In REM sleep, time-domain measures of HRV did not differ between OSA and no-OSA groups (AHI ≥ or < 5/h) (Table 3) in PAF patients. PAF patients with OSA showed increased parasympathetic modulation (HF-nu 32.6 ± 16.0 vs 40.5 ± 17.4, *p* = 0.036); however, significance was not maintained after adjusting for age, sex and BMI (Table 4). We extended the analysis to include OSA at different severity levels (AHI < or > 15/h, AHI < or > 30/h), although the above changes were not significant in these groups (see Tables S3, S4, S5 and S6 in the supplemental material).

### HRV correlations with markers of OSA

We examined correlations between HRV parameters and AHI, as well as other markers of OSA severity, including those that reflect hypoxic burden (ODI and %T < 90). In REM sleep, there was a weak negative correlation between LF-nu and all markers of OSA severity (AHI, ODI and %T < 90). Correlation analysis of other HRV (time and frequency) measures with OSA severity metrics were not significant (Supplementary material, Table S1 and S2).

In non-REM sleep, we saw a weak negative correlation between average NN interval and ODI as well as %T < 90. Correlations of other time and frequency HRV measures with markers of OSA severity (AHI, ODI and T < 90%) were not significant. (Supplementary material, Tables S1 and S2).

## Discussion

To our knowledge, this is the first study to compare HRV parameters in PAF patients with and without OSA. We found some evidence that PAF patients with OSA showed increased cardiac parasympathetic modulation (HF-nu) and blunted cardiac sympathetic modulation (LF-nu and LF/HF ratio) compared to PAF patients without OSA. The pathophysiological mechanism behind this finding needs further investigation but may provide future avenues for anti-arrhythmic therapeutic research. That these findings were limited to non-REM sleep is not surprising, given that REM sleep is a time of cardiovascular instability which may potentially mask differences in HRV between the groups.

Overall HRV (HRVi and total power) did not differ between PAF patients with and without OSA. Reduced overall HRV reflects a less adaptable ANS and is a strong independent predictor of mortality, in particular after myocardial infarction [14–22] and congestive heart failure [23–27]. Similarly, studies in AF patients show an association between depressed overall HRV and adverse outcome [28–33]. According to the Task Force of the European Society of Cardiology and the North American Society of Pacing Electrophysiology, a triangular index < 15 indicates a severely depressed sinus node activation. In our entire cohort of AF patients, the triangular index was, perhaps not surprisingly, well below this critical number (during non-REM and REM sleep), although it was similar between PAF patients with and without OSA.

Experimental studies indicate changes in the ANS and play a critical role in facilitating arrhythmic events and that concomitant modifiable risk factors such as OSA may further trigger AF [5]. Our short-term HRV analysis indicates there were no differences in overall HRV in PAF patients with and without OSA in any of the sleep stages. These results are in line with our recent systematic review that revealed nocturnal short-term measures of overall HRV were similar between patients with and without OSA [34] and therefore may extend to patient populations with PAF.

OSA events are well known to precipitate acute autonomic responses. For example, the initial apnoeic period is characterised by vagally-driven bradycardia, followed by a sympathetically-driven surge in heart rate and blood pressure with an accompanying arousal at the conclusion of the apnoeic event [35]. In this study, we deliberately excluded OSA events and the immediate post-apnoeic period (15 s) from the analysis in order to exclude the acute autonomic perturbations that accompany these events. This was done in order to compare chronic autonomic changes between the groups during a period of “steady state” sinus rhythm. Accordingly, we used short-term measures of HRV with a 2-min epoch. This was designed to maximise the availability of steady state ECG available for analysis, due to the frequency of excluded arrhythmic and respiratory events.

Additionally, particular anti-arrhythmic medications including Flecainide (class 1c) and β-blockers (class 11) are known to impact HRV through their effect on the ANS. For example, Flecainide has been shown to reduce HRV time–domain parameters [36]. In our study, the use of anti-arrhythmic medications in each individual class was not significantly different between the two groups, though the dosage and administration times were not measured. Furthermore, certain co-morbidities including acute myocardial infarction, diabetic neuropathy, heart transplantation and tetraplegia are known to significantly alter the function of the autonomic nervous system and hence HRV[36]. In our study, we corrected for the effect of age, sex and BMI. Most measured co-morbidities were not significantly different between groups, with the exception of hypertension, thyroid disease and peripheral vascular disease (see Table 2). Little is known about the influence of these particular conditions on HRV. However, one study demonstrated an increase in time domain and frequency domain–HRV parameters in AF patients with hypertension compared to patients with hypertension alone [37].

Several physiological studies demonstrated the importance of the autonomic nervous system in mediating sleep apnoea–induced AF. For example, vagal activation during the intra-thoracic pressure changes caused by acute apnoeic events shortens the atrial effective refractory period, thus increasing AF inducibility [38]. In a dog-model, Ghias et al. showed that after ablation of cardiac parasympathetic innervation, there was a significant decrease in apnoea-induced AF. This also occurred with sympatho-vagal blockade [39]. Similarly, Linz et al. showed in a pig model that the application of negative tracheal pressure induced AF via a shortening of the atrial refractory period and that this effect was negated by parasympathetic deactivation, either in the form of atropine administration or vagotomy [38]. During an acute obstructive apnoea, the profound vagal activation followed by combined sympathetic activation is thought to trigger and maintain AF. Experimental studies in chronic intermitted hypoxia show AF vulnerability and depends principally on parasympathetic activation; furthermore, parasympathetic activation has been identified as the major pro-arrhythmogenic mechanism in the rodent model [40]. Our data are line with this body of work, where PAF patients with OSA show increased parasympathetic modulation compared to PAF patients without OSA.

In addition to a major parasympathetic component, the sympathetic nervous system is also likely to contribute to AF promotion. However, in our study of PAF patients, we did not see elevated sympathetic modulation. Rather, our results suggest a blunted cardiac sympathetic modulation in PAF patients with OSA compared to PAF patients without OSA. This is somewhat surprising given that elevated sympathetic activity is well documented in OSA [34] and chronic intermittent hypoxia [41, 42]. However, one study in rats exposed to chronic intermittent hypoxia demonstrated elevated AF vulnerability that was accompanied by an elevated cholinergic response and damped beta-adrenergic response of the atrial myocardium [40]. It is possible that sympathetic activation maybe less important compared to parasympathetic activation in promoting AF due to elevated spatial dispersion of atrial refractoriness during parasympathetic activation [43]. Furthermore, the blunted sympathetic modulation in PAF patients with OSA in our study maybe associated with a ceiling effect driven by higher intrinsic adrenergic tone [40].

### Arrhythmia analysis

The study methodology provided an opportunity to compare the presence of nocturnal arrhythmia between AF patients with and without OSA, although this was not a primary aim of the study. On patient recall at interview, the patients in the OSA group reported a higher incidence of “high burden” AF, defined as ≥ 10 episodes in the past 12 months (8/36 patients (22%) vs 23/62 patients (37.1%), *p* = 0.039. On the sleep study night, there was no significant difference in % AF beats between the OSA and no-OSA groups, although the trend towards increased %AF beats in the OSA group was noted (2.9 ± 16.6 vs 8.1 ± 26.4%, *p* = 0.283. On subgroup analysis, however, the patients with severe OSA (AHI > 30/h) had more AF beats and more ventricular ectopic beats, but not supraventricular ectopic beats on nocturnal polysomnography. Similarly, PAF patients with severe OSA had a higher % AF beats than patients with no OSA or moderate OSA (mean difference 19.8 ± 7.3%, *p* = 0.040; 22.7 ± 8.3%, *p* = 0.036, respectively, Fig. 2). These findings of higher AF burden according to OSA severity are consistent with the findings of Mehra et al. [44], showing that nocturnal arrhythmia including AF and VEBs were more common in patients with severe sleep-disordered breathing, also using a cut-off of AHI > 30/h. To our knowledge, our study is the first to replicate this finding in a cohort of patients with PAF with and without OSA.

### Limitations

Although this study provides some novel insights into the HRV profile of those with OSA and PAF, there are limitations to the study. Twenty-four hour Holter recording is ideal for HRV analysis, accounting for both diurnal and nocturnal variability [7]. For this study, HRV parameters were derived from nocturnal polysomnograpy and thus are subject to all the usual autonomic perturbations of sleep, which may explain the selective differences seen across time and frequency-domain measures. Furthermore, we were unable to control for differences in undiagnosed conduction disturbances between the two groups, including, for example, the presence of sinus nodal disease which has a high prevalence in AF patients [45]. However, we analysed only periods of sinus rhythm in order to minimise the contribution of AV nodal dysfunction. Our study contained some patients who had undergone previous PVI: patients had, on average, undergone 0.4 ± 0.6 previous PVI procedures. Since PVI may cause neuronal damage to the intrinsic cardiac nervous system [46], caution must be used when extrapolating the results to other groups. Although we excluded periods of arrhythmia, it is possible that autonomic disturbances related to the arrhythmia may have preceded or persisted beyond these events. It is also possible that HRV parameters may have been impacted by autonomic disruptions from acute obstructive respiratory events in the OSA group. We attempted to allow for this by excluding ECG trace during and immediately following sleep apnoea events from the analysis; however, we excluded a post-event period of 15 s, and it is possible autonomic disturbance may persist beyond this interval. In addition, some sub-criterion respiratory events are likely to have remained in the analysis.

### Clinical implications

There is mounting evidence that the perturbations during OSA have a profound influence on the myocardium [6]. Atrial remodelling leading to changes to the electrical conduction and ANS activation is thought to trigger and maintain AF [6]. Our work shows altered autonomic function in PAF patients with co-morbid OSA which we believe supports previous observations that progression of AF is promoted by the presence of modifiable risk factors such as OSA [5, 6]. Treatment of OSA may modulate autonomic function and protect the atrial myocardium from pro-arryhthmic autonomic influences from OSA. Therefore, future studies should look to replicate our findings in a larger cohort and determine the effect of OSA therapy on modulation of the ANS and whether indeed such interventions may mitigate arrhythmogenesis in PAF.

## Conclusions

This is the first study to compare sympatho-vagal balance, assessed by HRV, in PAF patients with and without OSA. Our results indicate limited differences in HRV between groups. However, we found some evidence of increased parasympathetic modulation and decreased sympathetic modulation in the OSA group. Altered autonomic function in this group may promote arrhythmogenesis and impair antiarrhythmic therapy. Elucidating influence of OSA on autonomic function in patients with AF may inform treatment strategies that mitigate pro-arrhythmic autonomic influences. Future studies should look to replicate our findings in a larger cohort and determine the effect of OSA therapy on modulation of the ANS.

## Supplementary Information

Below is the link to the electronic supplementary material.

Supplementary file1 (DOCX 39 KB)
