# Supplementary material for: Does obstructive sleep apnoea modulate cardiac autonomic function in paroxysmal atrial fibrillation?
Source: J Interv Card Electrophysiol. 2022 Apr 9;66(4):873–83. doi: 10.1007/s10840-022-01202-3 (PMC10172286; doi:10.1007/s10840-022-01202-3)
Supplement: Supplementary file 1 — Supplementary file1 (DOCX 39 KB) [file 10840_2022_1202_MOESM1_ESM.docx]

**Does obstructive sleep apnoea modulate Cardiac Autonomic Function in Paroxysmal Atrial Fibrillation?**

**Supplementary Material**

**TABLE S1: HRV Time-Domain Pearson correlations with OSA parameters**

|  | PSG AHI |  | PSG ODI |  | %T<90 |  |
| --- | --- | --- | --- | --- | --- | --- |
|  | R value | P value | R value | P value | R value | P value |
| **Average NN interval** | | | | | | |
| Non-REM | -0.169 | 0.112 | -.215 | 0.043* | -.211^*^ | 0.047* |
| REM | -0.095 | 0.386 | -0.151 | 0.167 | -0.160 | 0.140 |
| † **RMSSD** | | | | | | |
| Non-REM | 0.043 | 0.692 | 0.032 | 0.768 | 0.087 | 0.415 |
| REM | 0.137 | 0.207 | 0.099 | 0.366 | 0.101 | 0.354 |
| † **pNN50** | | | | | | |
| Non-REM | 0.104 | 0.336 | 0.082 | 0.450 | 0.050 | 0.644 |
| REM | -0.065 | 0.551 | -0.110 | 0.312 | -0.008 | 0.943 |
| **Triangular Index** | | | | | | |
| Non-REM | -0.022 | 0.841 | -0.048 | 0.658 | 0.008 | 0.942 |
| REM | -0.017 | 0.878 | -0.038 | 0.727 | 0.006 | 0.957 |

Natural log-transformed data are indicated by †

PSG: polysomnogram, AHI: Apnea Hypopnea Index, ODI: Oxygen Desaturation Index, %T<90: percent of sleep time with haem-oxygen saturation < 90%, Non-REM: non-rapid eye movement; REM: rapid eye movement; Average NN interval: average of N wave to N wave variation, RMSSD: square root of the mean squared differences of successive NN intervals, pNN50: percentage of successive NN intervals that differ by more than 50ms, Triangular Index: integral of the density of the RR interval histogram divided by its height.

**TABLE S2: HRV Frequency-Domain Pearson correlations with OSA parameters**

|  | **PSG AHI** |  | **PSG ODI** |  | **%T<90** | |  |
| --- | --- | --- | --- | --- | --- | --- | --- |
|  | R value | P value | R value | P value | R value | | P value |
| † **High Frequency** | | | | | | | |
| Non-REM | -0.001 | 0.993 | 0.001 | 0.995 | 0.128 | | 0.233 |
| REM | 0.122 | 0.262 | 0.090 | 0.409 | 0.095 | | 0.386 |
| **High Frequency Normalised Units** | | | | | | | |
| Non-REM | 0.036 | 0.738 | 0.082 | 0.443 | 0.059 | | 0.582 |
| REM | 0.182 | 0.094 | 0.190 | 0.079 | 0.114 | | 0.296 |
| †**Low Frequency** | | | | | | | |
| Non-REM | -0.063 | 0.559 | -0.084 | 0.435 | 0.060 | | 0.579 |
| REM | -0.017 | 0.876 | -0.041 | 0.708 | -0.011 | | 0.917 |
| **Low Frequency Normalised Units** | | | | | | | |
| Non-REM | -0.141 | 0.187 | -0.192 | 0.071 | -0.164 | | 0.125 |
| REM | -.219 | 0.043* | -.219 | 0.042* | -.215 | | 0.046* |
| †**Very Low Frequency** | | | | | | | |
| Non-REM | -0.095 | 0.377 | -0.101 | 0.344 | -0.006 | | 0.954 |
| REM | -0.071 | 0.518 | -0.056 | 0.608 | -0.021 | | 0.845 |
| **LF/HF Ratio** | | | | | | | |
| Non-REM | -0.083 | 0.437 | -0.106 | 0.323 | -0.102 | | 0.341 |
| REM | -0.143 | 0.188 | -0.134 | 0.217 | -0.109 | | 0.317 |
| † **Total Power** | | | | | | | |
| Non-REM | -0.028 | 0.793 | -0.037 | 0.730 | 0.094 | 0.379 | |
| REM | 0.017 | 0.875 | -0.004 | 0.969 | 0.020 | 0.857 | |

Natural log-transformed data are indicated by †

PSG: polysomnogram, AHI: Apnea Hypopnea Index, ODI: Oxygen Desaturation Index, %T<90: percent of sleep time with haem-oxygen saturation less than 90%; Non-REM: non-rapid eye movement, REM: rapid eye movement, High frequency: Power in the 0.15-0.40Hz band, High frequency normalised units: HF power divided by power ≥0.04Hz, Low Frequency: Power in the 0.04-0.15Hz band, Low Frequency normalised units: LF power divided by power ≥0.04Hz, Very low frequency: Power less than 0.04Hz, LF/HF, Low frequency/High frequency ratio: Low frequency power divided by High frequency power, Total Power: Power from 0 to Nyquist frequency.

**TABLE S3: HRV Time-Domain Parameters by OSA Status (AHI < or ≥15/hr) in a cohort with Atrial Fibrillation**

|  | **All patients** | **No OSA/Mild OSA**  **AHI < 15/hr** | **Moderate to Severe OSA**  **AHI ≥ 15/hr** | **P value** | **P value (adjusted for age, sex, BMI)** | | |
| --- | --- | --- | --- | --- | --- | --- | --- |
|  | **n = 89** | **n = 64** | **n = 25** |  |  |  |  |
| **Average NN interval (ms)** | | | | | |  |  |
| Non-REM | 1080.2 ± 162.9 | 1097.6 ± 166.5 | 1035.7 ± 147.1 | 0.053 | 0.074 |  |  |
| REM | 1067.2 ± 166.4 | 1080.9 ±169.6 | 1029.9 ± 154.7 | 0.105 | 0.076 |  |  |
| †**RMSSD (ms)** | | | | | |  |  |
| Non-REM | 28.7 (23.1) | 28.3 (38.5) | 30.9 (20.7) | 0.231 | 0.180 | |  |
| REM | 26.7 (22.7) | 22.8 (24.0) | 23.4 (19.5) | 0.375 | 0.616 | |  |
| †**pNN50 (%)** | | | | | |  |  |
| Non-REM | 5.5 (14.7) | 5.6 (18.7) | 4.8 (13.6) | 0.369 | 0.827 | |  |
| REM | 2.8 (12.3) | 2.9 (14.4) | 2.8 (6.0) | 0.211 | 0.351 | |  |
| **Triangular Index (nu)** | | | | | |  |  |
| Non-REM | 10.8 ± 4.3 | 11.1 ± 4.4 | 9.9 ± 4.2 | 0.135 | 0.162 | |  |
| REM | 11.7 ± 4.6 | 12.1 ± 4.6 | 10.6 ± 4.8 | 0.093 | 0.170 | |  |

Data are presented as mean ± SD or median (IQR). Natural log-transformed data are indicated by †

Non-REM: non-rapid eye movement; REM: rapid eye movement; Average NN interval: average of N wave to N wave variation, RMSSD: square root of the mean squared differences of successive NN intervals, pNN50: percentage of successive NN intervals that differ by more than 50ms, Triangular Index: integral of the density of the RR interval histogram divided by its height.

**TABLE S4: HRV Frequency-Domain Parameters by OSA Status (AHI < or ≥15/hr) in a cohort with Atrial Fibrillation**

|  | **All patients** | **No OSA/Mild OSA**  **AHI < 15/hr** | | **Moderate to Severe OSA**  **AHI ≥15/hr** | | **P value** | | **P value (adjusted for age, sex, BMI)** | | |
| --- | --- | --- | --- | --- | --- | --- | --- | --- | --- | --- |
|  | **n = 89** | **n = 64** | | **n = 25** | |  | |  | |  |
| † **High Frequency (ms^2^)** | | | | | | | | | | |
| Non-REM | 333.3 (437.8) | 314.6 (1116.8) | | 396.7 (570.8) | | 0.320 | | 0.111 | |  |
| REM | 223.3 (437.8) | 217.4 (421.4) | | 225.8 (491.4) | | 0.635 | | 0.554 | |  |
| **High Frequency, Normalised Units (%)** | | | | | | | | | | |
| Non-REM | 44.5 ± 15.6 | 43.7 ± 15.0 | | 46.5 ± 17.1 | | 0.463 | | 0.889 | |  |
| REM | 37.4 ± 17.2 | 35.6 ± 16.4 | | 42.3 ± 18.8 | | 0.111 | | 0.388 | |  |
| † **Low Frequency (ms^2^)** | | | | | | | | | | |
| Non-REM | 336.1 (863.9) | 354.9 (1251.8) | | 307.6 (369.7) | | 0.095 | | 0.063 | |  |
| REM | 302.9 (817.5) | 337.9 (1118.6) | | 289.9 (630.5) | | 0.126 | | 0.127 | |  |
| **Low Frequency Normalised Units (%)** | | | | | | | | | | |
| Non-REM | 47.8 ± 19.2 | 49.8 ± 17.7 | | 42.6 ± 22.3 | | 0.116 | | 0.457 | |  |
| REM | 55.2 ± 22.1 | 58.0 ± 20.5 | | 47.6 ± 25.0 | | 0.054 | | 0.150 | |  |
| †**Very Low Frequency (ms^2^)** | | | | | | | | | | |
| Non-REM | 470.0 (860.0) | 505.2 (1076.6) | | 439.4 (518.0) | | 0.072 | | 0.031 | |  |
| REM | 865.4 (1322.0) | 1019.3 (1508.0) | | 618.8 (1240.9) | | 0.036* | | 0.023* | |  |
| **LF/HF Ratio (nu)** | | | | | | | | | | |
| Non-REM | 1.5 ± 1.5 | 1.6 ± 1.6 | | 1.3 ± 1.2 | | 0.426 | | 0.996 | |  |
| REM | 2.3 ± 2.2 | 2.5 ± 2.3 | | 1.9 ± 2.1 | | 0.285 | | 0.562 | |  |
| † **Total Power** | | | | | | | | | |  |
| Non-REM | 1352.7 (2078.9) | | 1375.5 (3401.3) | | 1363.2 (1315.0) | | 0.191 | | 0.079 |  |
| REM | 1128.2 (1498.7) | | 1610.6 (2946.8) | | 1442.6 (2010.1) | | 0.199 | | 0.181 |  |

**TABLE S5: HRV Time-Domain Parameters by OSA Status (AHI < or ≥30/hr) in a cohort with Atrial Fibrillation**

|  | **All patients** | **AHI < 30/hr** | **Severe OSA**  **AHI ≥ 30/hr** | **P value** | **P value (adjusted for age, sex, BMI)** | | |
| --- | --- | --- | --- | --- | --- | --- | --- |
|  | **n = 89** | **n = 81** | **n = 8** |  |  |  |  |
| **Average NN interval (ms)** | | | | | |  |  |
| Non-REM | 1080.2 ± 162.9 | 1092.1 ± 157.1 | 959.9 ± 182.7 | 0.028* | 0.018* |  |  |
| REM | 1067.2 ± 166.4 | 1075.5 ± 161.5 | 974.3 ± 205.2 | 0.124 | 0.056 |  |  |
| †**RMSSD (ms)** | | | | | |  |  |
| Non-REM | 28.7 (23.1) | 29.6 (27.2) | 26.7 (42.2) | 0.732 | 0.799 | |  |
| REM | 26.7 (22.7) | 22.8 (22.7) | 25.5 (108.2) | 0.298 | 0.396 | |  |
| †**pNN50 (%)** | | | | | |  |  |
| Non-REM | 5.5 (14.7) | 5.7 (15.0) | 3.5 (6.4) | 0.555 | 0.888 | |  |
| REM | 2.8 (12.3) | 2.9 (13.5) | 2.6 (3.7) | 0.392 | 0.220 | |  |
| **Triangular Index (nu)** | | | | | |  |  |
| Non-REM | 10.8 ± 4.3 | 10.7 ± 4.4 | 11.0 ± 3.6 | 0.876 | 0.594 | |  |
| REM | 11.7 ± 4.6 | 11.6 ± 4.6 | 11.8 ± 5.7 | 0.936 | 0.631 | |  |

Data are presented as mean ± SD or median (IQR). Natural log-transformed data are indicated by †

Non-REM: non-rapid eye movement; REM: rapid eye movement; Average NN interval: average of N wave to N wave variation, RMSSD: square root of the mean squared differences of successive NN intervals, pNN50: percentage of successive NN intervals that differ by more than 50ms, Triangular Index: integral of the density of the RR interval histogram divided by its height.

**TABLE S6: HRV Frequency-Domain Parameters by OSA Status (AHI < or ≥30/hr) in a cohort with Atrial Fibrillation**

|  | **All patients** | **No OSA/Mild OSA**  **AHI < 30/hr** | | **Severe OSA**  **AHI > 30/hr** | | **P value** | **P value (adjusted for age, sex, BMI)** | | |
| --- | --- | --- | --- | --- | --- | --- | --- | --- | --- |
|  | **n = 89** | **n = 81** | | **n = 8** | |  |  | |  |
| † **High Frequency (ms^2^)** | | | | | | | | | |
| Non-REM | 333.3 (437.8) | 321.2 (703.4) | | 355.6 (1345.8) | | 0.642 | 0.664 | |  |
| REM | 223.3 (437.8) | 217.4 (386.6) | | 401.5 (33331.1) | | 0.236 | 0.354 | |  |
| **High Frequency, Normalised Units (%)** | | | | | | | | | |
| Non-REM | 44.5 ± 15.6 | 44.9 ± 15.2 | | 40.1 ± 19.9 | | 0.405 | 0.081 | |  |
| REM | 37.4 ± 17.2 | 37.0 ± 16.8 | | 42.0 ± 22.5 | | 0.466 | 0.481 | |  |
| † **Low Frequency (ms^2^)** | | | | | | | | | |
| Non-REM | 336.1 (863.9) | 319.2 (989.3) | | 342.3 (780.2) | | 0.953 | 0.890 | |  |
| REM | 302.9 (817.5) | 301.2 (830.9) | | 328.3 (836.6) | | 0.610 | 0.851 | |  |
| **Low Frequency Normalised Units (%)** | | | | | | | | | |
| Non-REM | 47.8 ± 19.2 | 47.9 ± 18.5 | | 46.6 ± 27.3 | | 0.861 | 0.624 | |  |
| REM | 55.2 ± 22.1 | 56.1 ± 21.6 | | 45.5 ± 26.8 | | 0.229 | 0.133 | |  |
| †**Very Low Frequency (ms^2^)** | | | | | | | | | |
| Non-REM | 470.0 (860.0) | 493.6 (877.5) | | 374.8 (628.4) | | 0.971 | 0.648 | |  |
| REM | 865.4 (1322.0) | 890.5 (1310.2) | | 618.8 (815.0) | | 0.809 | 0.835 | |  |
| **LF/HF Ratio (nu)** | | | | | | | | | |
| Non-REM | 1.5 ± 1.5 | 1.5 ± 1.6 | | 1.6 ± 1.2 | | 0.807 | 0.293 | |  |
| REM | 2.3 ± 2.2 | 2.4 ± 2.2 | | 1.9 ± 2.2 | | 0.608 | 0.481 | |  |
| † **Total Power** | | | | | | | | |  |
| Non-REM | 1352.7 (2078.9) | | 1374.6 (2329.6) | | 1573.3 (2862.1) | 0.744 | | 0.937 |  |
| REM | 1128.2 (1498.7) | | 1576.9 (2573.7) | | 1664.9 (6913.2) | 0.470 | | 0.663 |  |

Data are presented as mean (SD) or median (IQR). Natural log-transformed data are indicated by †

High frequency: Power in the 0.15-0.40Hz band, High frequency normalised units: HF power divided by power ≥0.04Hz, Low Frequency: Power in the 0.04-0.15Hz band, Low Frequency normalised units: LF power divided by power ≥0.04Hz Very low frequency: Power less than 0.04Hz, LF/HF, Low frequency/high frequency ratio: Low frequency power divided by high frequency power, Total Power: Power from 0 to Nyquist frequency
